# Harnessing Silver Nanoclusters to Combat *Staphylococcus aureus* in the Era of Antibiotic Resistance

**DOI:** 10.3390/pharmaceutics17030393

**Published:** 2025-03-20

**Authors:** Julieta Chiappero, Gustavo A. Monti, Diego F. Acevedo, Natalia S. Paulucci, Edith I. Yslas

**Affiliations:** 1 Departamento de Biología Molecular, Facultad de Ciencias Exactas, Físico-Químicas y Naturales, Instituto de Biotecnología Ambiental y Salud, INBIAS, CONICET-UNRC, Universidad Nacional de Río Cuarto, Río Cuarto 5800, Argentina; jchiappero@exa.unrc.edu.ar; 2 Departamento de Tecnología Química, Facultad de Ingeniería, Instituto de Investigaciones en Tecnologías Energéticas y Materiales Avanzados, IITEMA, CONICET-UNRC, Universidad Nacional de Río Cuarto, Río Cuarto 5800, Argentina; gmonti@exa.unrc.edu.ar (G.A.M.); dacevedo@ing.unrc.edu.ar (D.F.A.); 3 Departamento de Química, Facultad de Ciencias Exactas Físico-Químicas y Naturales, Instituto de Investigaciones en Tecnologías Energéticas y Materiales Avanzados, IITEMA, CONICET-UNRC, Universidad Nacional de Río Cuarto, Río Cuarto 5800, Argentina; 4 Departamento de Biología Molecular, Facultad de Ciencias Exactas, Físico-Químicas y Naturales, Instituto de Investigaciones en Tecnologías Energéticas y Materiales Avanzados, IITEMA, CONICET-UNRC, Universidad Nacional de Río Cuarto, Río Cuarto 5800, Argentina

**Keywords:** silver nanoclusters, eco-friendly synthesis, stabilizing polymers, bacterial infections, *Staphylococcus aureus*, antimicrobial activity, hemocompatibility

## Abstract

**Background/Objectives**: In the race to develop new antibiotics to combat multidrug-resistant bacteria, particularly the *ESKAPE* pathogens which pose a significant threat to public health, silver nanoclusters (AgNCs) have emerged as a promising alternative. This article focuses on the potential of novel silver nanoclusters as an antimicrobial agent against *Staphylococcus aureus*, a high-priority pathogen known for its ability to cause persistent nosocomial infections and develop protective biofilms. **Methods**: In this study, we successfully synthesized AgNCs at pH 7 using an eco-friendly photoreduction method with poly acrylic acid (PAA) and poly methacrylic acid (PMAA) as stabilizers. This methodology produced fluorescent AgNCs, demonstrating their stability in aqueous solutions for at least three months and highlighting the effectiveness of PAA and PMAA as stabilizing agents. The AgNCs were incubated with *S. aureus* suspension, and the antimicrobial capability at different concentrations and times of incubation were determined. Also, the AgNCs hemocompatibility was studied by exposing the clusters to rat blood cells. **Results**: The in vitro assays revealed that AgNCs capping with PAA or PMAA has antimicrobial activity in low doses (the determination of minimum inhibitory concentration (MIC): 0.2 µg/mL, and the determination of minimum bactericidal concentration (MBC): 2 µg/mL) and without cytotoxicity (hemolysis less than 10%) to rat blood cells until 1 µg/mL. In the presence of both AgNCs (5 µg/mL), bacterial growth was completely inhibited within just 3 h. **Conclusions:** The findings of this study highlight the potential of silver nanoclusters as effective antimicrobial agents against *S. aureus*. Their stability, low toxicity, and rapid bactericidal activity make them promising candidates for further development in antimicrobial applications.

## 1. Introduction

Since the discovery of antibiotics, they have been used indiscriminately and inadequately, leading to the present situation where there has been a surge in multidrug-resistant bacteria. These superbugs are found to cause hospital-acquired infections and are part of a group known as the *ESKAPE* (*Enterococcus faecium*, *Staphylococcus aureus*, *Klebsiella pneumoniae*, *Acinetobacter baumannii*, *Pseudomonas aeruginosa* and *Enterobacter* spp.) [1,2]. *ESKAPE* bacteria are currently under high consideration by the health system because of their highly infective nature and their influence on mortality and morbidity. Gram-positive bacteria such as *S. aureus* (opportunistic pathogen) are high-priority pathogens in the World Health Organization (WHO) list due to their ability to cause a variety of nosocomial infections in patients with compromised immune systems [3,4]. These infections can range from skin, soft tissues, and osseous tissues to pleuropulmonary infections, pneumonia and endocarditis, and bacteremia or septicemia, and can also affect medical devices such as prostheses, catheters, or other plastic materials [3,5,6]. The multidrug resistance exhibited by *S. aureus* is mainly due to its ability to produce biofilm, protecting microorganisms against the immune system and antimicrobials [5], presenting a challenge to the development of effective antibiotic therapies. Of the infections caused by *S. aureus*, almost 50% are caused by methicillin-resistant *S. aureus* (MRSA). MRSA is associated with high mortality and morbidity due to limited antibiotic treatment options. For the treatment of this type of infection, vancomycin is used as a first-line treatment [7]. The 2011 Infectious Diseases Society of America (IDSA) guidelines [8] recommend vancomycin through levels of less than 15 mg/L for mild MRSA infections and up to a maximum of 15–20 mg/L for severe cases since prolonged exposure to higher concentrations of this antibiotic generates nephrotoxicity [7].

In recent years, alternative approaches to the exclusive use of antibiotics have been developed. The advance of nanotechnology has opened the door to studying nanomaterials with antibacterial capacities. Metals including silver, zinc and copper have been used in medicine for infection control due to their antibacterial properties [9]. Silver nanoparticles are widely used as antimicrobials as a result of their effectiveness against bacteria, viruses, and fungi in a variety of applications. Also, it can be used as disinfectant and shows synergistic effect when applied with antibiotics [10].

The silver-based antibacterial system, as previously mentioned, represents a highly promising antibacterial material. Nonetheless, it is crucial to continuously enhance its antimicrobial efficacy to optimize bactericidal activity. Silver nanoclusters (AgNCs) are nanoscale assemblies composed of a few to several dozen silver atoms. In recent years, AgNCs have garnered significant attention due to their unique properties and diverse applications across various fields [11]. Typically ranging in size from a sub-nanometer to a few nanometers, AgNCs exhibit distinctive electronic and optical features that set them apart from silver nanoparticles or bulk silver. This quantum confinement effect results in tunable fluorescence, allowing these clusters to emit light in the visible and near-infrared regions [12].

The potent antimicrobial effect is given by the dispersion of silver ions (Ag^+^) with the consequent effect of cellular membrane disruption, DNA replication interruption, and the accumulation of reactive oxygen species (ROS) that lead to a cellular oxidative burst and death [13]. AgNCs are generally unstable because atomic silver (Ag^0^) is highly reactive (oxidation susceptibility), which is a major challenge in exploiting their full potential. In this sense, stabilizing agents are essential to prevent aggregation and maintain the structural integrity of AgNCs. Among the various stabilization strategies, polymeric matrices such as poly methacrylic acid (PMAA) and polyacrylic acid (PAA) are widely recognized for their exceptional stabilizing properties, improving their stability and prolonging their lifetime, and for being biocompatible [14].

Although the potential of AgNCs as antibacterial agents has been demonstrated [15,16,17,18], it is dependent on unique properties such as size, surface characteristics, and stability [17].

In this work, we used two stabilizing polymers to obtain small-sized AgNCs using an environmentally friendly system with high antimicrobial capacity against *S. aureus* at low doses. The MIC did not show a hemolytic effect on red blood cells, and it was significantly lower than the vancomycin MIC (conventional antibiotic) determined in this study.

## 2. Materials and Methods

### 2.1. Synthesis of Ag-PAA NCs and Ag-PMAA NCs

The synthesis of water-dispersible fluorescence Ag-PAA NCs and Ag-PMAA NCs was performed by ultraviolet photoreduction. This procedure involves a reduction reaction induced by the irradiation of light. In comparison to other methods, this is a green, non-toxic, and less time-consuming approach which does not require any additional reducing agent. Aqueous solutions of polyacrylic acid sodium salt (PAA, Sigma-Aldrich (St. Louis, MO, USA), Mw = 15000) and poly methacrylic acid sodium salt (PMAA, Sigma-Aldrich (St. Louis, MO, USA), Mw = 9500) with a concentration of 5 mg/mL were prepared. The stock solutions of PAA and PMAA were mixed with the metal precursor AgNO_3_ (Sigma-Aldrich (St. Louis, MO, USA), >99.8%) to obtain a solution with a concentration ratio of Ag/stabilizer of 1:1 per unit mass.

The pH of the solutions was adjusted to 7 using 0.1 M HClO_4_ (Cicarelli (San Lorenzo, Santa Fe, Argentina), 70%) for cluster formation. These solutions were then irradiated with a UV lamp (365 nm, 32 W) for 1 h to photo-reduce all Ag^+^ and obtain Ag-PAA NCs and Ag-PMAA NCs. The AgNCs were stored at 4 °C in the dark.

### 2.2. Ag-PAA NC and Ag-PMAA NC Characterization

UV-vis spectra were recorded using a spectrophotometer Shimadzu 2401 (Kioto, Prefectura de Kioto, Japan) with a sample holder with a thermostat at 25 °C.

A Fluoromax Horiba Plus spectrofluorometer (Kioto, Prefectura de Kioto, Japan) was employed to analyze the fluorescent properties of the AgNCs.

### 2.3. Microorganisms and Treatments

The microorganism under study was *Staphylococcus aureus* (ATCC 25923). *S. aureus* was kept in glycerol at −80 °C. For the assays, the strain was cultivated in Luria Bertani (LB) Agar at 37 °C for 24 h. A single colony was transferred to LB broth and incubated in a shaker with a thermostat at 37 °C for 24 h. An aliquot of this inoculum was placed in fresh LB broth and incubated in a shaker with a thermostat at 37 °C for 3 h to obtain the bacterial culture in the exponential phase.

The bacterial assays performed in this work were carried out using PBS (phosphate salt buffer) as diluent to achieve 1 × 10^6^ CFU/mL. The following treatments were established: C (control *S. aureus*); P* (pure PAA 5 μg/mL); P** (pure PMAA 5 μg/mL); T1 (AgNCs 0.1 μg/mL + *S. aureus*); T2 (AgNCs 0.2 μg/mL + *S. aureus*); T3 (AgNCs 1 μg/mL + *S. aureus*); T4 (AgNCs 2 μg/mL + *S. aureus*); and T5 (AgNCs 5 μg/mL + *S. aureus*). Individual treatments were performed separately for every AgNC, which were incubated in a shaker at 37 °C for 3 h.

### 2.4. Determination of Minimum Inhibitory Concentration (MIC) of Ag-PAA NCs and Ag-PMAA NCs

**Figure 1:**
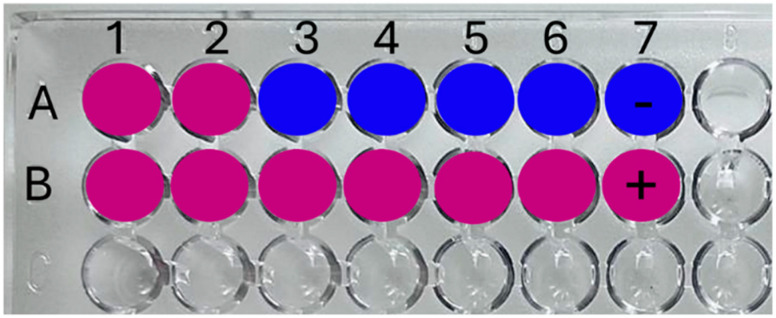
Schematic disposition of samples and controls in the microtiter plate (96 wells) for the resazurin technique. (**A**) (1–6) Different AgNC concentrations and (7) negative technique control; (**B**) (1–6) Different polymer concentrations and (7) positive technique control.

The antimicrobial activity of the AgNCs was determined using the method described by Mann and Markham [19] with modifications. Resazurin sodium salt served as a redox indicator, appearing blue when oxidized and pink when reduced. It was used as a marker of active metabolism in bacterial cultures among other uses. Several mechanisms for resazurin reduction by viable cells have been described, utilizing NADH and NADPH as electron sources. These mechanisms include reduction by mitochondrial or microsomal enzymes, enzymes in the respiratory chain, or electron transfer agents such as N-methylphenazinium methosulfate [20]. The microorganism cell concentration required to reduce resazurin within 3 h was determined. Serial 10-fold dilutions of the overnight culture were prepared in PBS (1 × 10^−2^; 1 × 10^−3^; 1 × 10^−4^; 1 × 10^−5^; 1 × 10^−6^). Aliquots (170 μL) of each dilution were dispensed into a microtiter plate containing 20 μL of PBS and 10 μL of resazurin solution (0.2% w/v). The microtiter plate was incubated for 3 h at 37 °C. The appropriate dilution was the first where resazurin was not reduced (blue), and it was selected to carry out the following tests (1 × 10^−4^ which corresponds to 1 × 10^6^ CFU/mL). Subsequently, a sterile 96-well microtiter tray was set up as follows (Figure 1): in row A, from column 1 to 6, 170 μL inoculum + 20 μL of each AgNC concentration (0; 0.1; 0.2; 1; 2; and 5 μg/mL); column 7-A: 190 μL assay medium (PBS) (negative control); column 7-B: 170 μL inoculum + 20 μL of diluent PBS (positive control). Next, 10 μL of resazurin solution was added to all the wells. The microtiter plate was incubated at 37 °C for 3 h in dark conditions. After incubation, the wells were assessed visually for color change, with the highest dilution remaining blue, indicating the MIC. This assay was performed also with the PAA and PMAA stock solution polymers to discard equal intrinsic-effect-testing concentrations in row B, columns 1 to 6. The same experiment was performed with the commercial antibiotic vancomycin in a range of concentrations (0.5; 1; 2; 5; 10; 32; and 100 μg/mL). The dose ranges were based on guidelines established by the CLSI (Clinical and Laboratory Standards Institute) and the SFM (French Society for Microbiology), which established a sensible vancomycin microorganism when the MIC is less than 4 μg/mL, the intermedial sensibility to an MIC between 8 to 16 μg/mL, and a resistant microorganism when the vancomycin MIC is over 32 μg/mL [21].

### 2.5. Determination of Minimum Bactericidal Concentration (MBC) of Ag-PAA NCs and Ag-PMAA NCs

The MBC was determined as follows: 100 µL of the dilution corresponding to the MIC and its subsequent dilutions were cultivated in LB agar and incubated at 37 °C for 24 h. The MBC was defined as the last dilution without cellular growth [22].

### 2.6. Cell Growth Inhibitory Assay “In Vitro”

An aliquot (1 × 10^6^ CFU/mL) of *S. aureus* culture in the exponential phase was incubated in PBS with each cluster (Ag-PAA NCs and Ag-PMAA NCs); treatments are described in “treatments section”. The control was incubated under equal conditions, with no nanoclusters. This assay was performed also with the PAA and PMAA pure polymers (5 μg/mL) to discard the intrinsic effect. After the incubation time, we proceeded to conduct the colony counting using the microdrop technique [23].

### 2.7. AgNCs on Bacterial Death Kinetics

An aliquot of *S. aureus* culture in the exponential phase (1 × 10^6^ CFU/mL) was incubated in PBS with 0.2 μg/mL or 5 μg/mL of each AgNC at 37 °C for 48 h with shaking. The control was incubated under equal conditions, without AgNCs. Colony counts were determined using the microdrop technique [23] at different time points (0; 0.25; 1; 3; 24; and 48 h). The results were compared with the control with no AgNCs.

### 2.8. Hemolysis Assay “In Vitro”

The hemolysis assay was proposed following the Guo et al. [2] method with modifications. The experiments were conducted with blood samples of young female Wistar rats donated by investigators of Universidad Nacional de Río Cuarto in accordance with the guidelines of the Comité de Ética de la Investigación (COEDI) at Universidad Nacional de Río Cuarto. Samples (1 mL) were collected using heparin as an anticoagulant (Sodium heparin 5000 U.I./mL; Veinfar, (Buenos Aires, Argentina)). After that, 2 mL of PBS was added to this tube, and the red cells were isolated from the serum by centrifugation at 1000 rpm for 10 min. After obtaining the isolated red cells, they were washed three times with 5 mL of PBS solution. An aliquot of 250 μL of red cells was placed in Eppendorf-like microtubes, with 250 μL of each AgNC concentration (0.05; 0.1; 0.2; 0.5; 1; and 5 μg/mL). The samples were incubated for 3 h at 37 °C, then the cell suspensions were centrifuged at 1000 rpm for 5 min. Aliquots (200 μL) of the supernatant were transferred to a 96-well microtiter plate, and the corresponding hemoglobin release was monitored at 576 nm using a microplate reader (Thermo Scientific, Multiskan FC, Waltham, MA, USA). The red cell suspension with PBS was used as a negative control, and the absorbance of red cells lysed by distilled water was taken as 100% hemolysis. The same assay was performed also with PAA and PMAA polymers to discard the intrinsic effect.

### 2.9. Statistical Analyses

Data was subjected to an analysis of variance (ANOVA) followed by a comparison of multiple treatment levels using Tukey’s post hoc test. Differences between means were significant at *p* < 0.0001. Statistical analyses were performed with InfoStat software version 2020.

## 3. Results and Discussion

### 3.1. Ag-PAA NC and Ag-PMAA NC Characterizations

**Figure 2:**
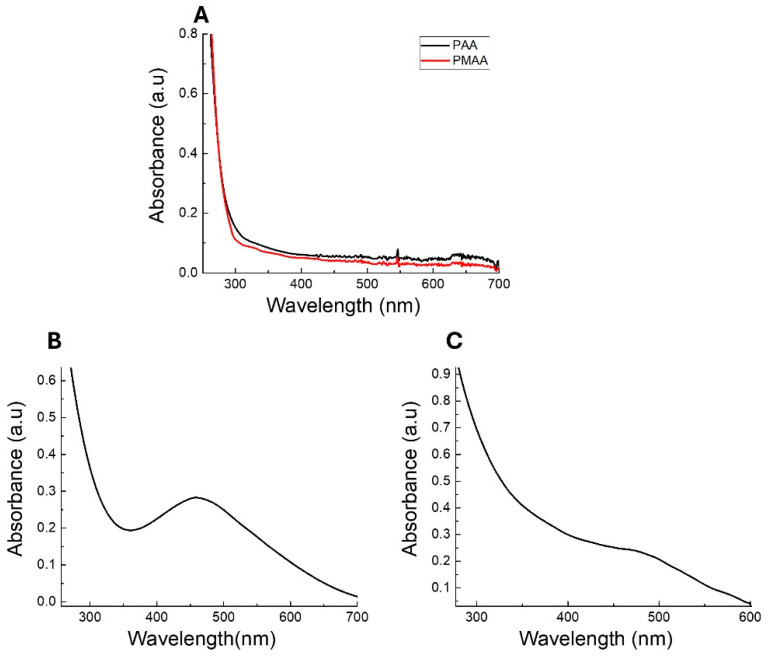
UV-vis spectra of solution pH 7 of (**A**) polymer PAA (black line) and PMAA (red line); (**B**) Ag-PAA NCs; and (**C**) Ag-PMAA NCs.

The optical properties of the obtained AgNCs were studied. Figure 2 shows the absorption spectra of the polymers, Ag-PAA NCs, and Ag-PMAA NCs in solution at pH 7. It should be noted that both polymers exhibited intense adsorption below 350 nm and that the adsorption decreased considerably at longer wavelengths (Figure 2A). As can be seen, the Ag-PAA NCs (Figure 2B) showed a band with an adsorption maximum around 460 nm. Likewise, the spectrum of Ag-PMAA NCs (Figure 2C) showed intense absorption at 475 nm. Ag-PAA NC and Ag-PMAA NC spectra presented significant differences from the polymers (Figure 2A). The absorption band in the spectral range between 450 and 480 nm has been attributed by several authors to the presence of AgNCs with sizes ranging from 2 nm to 4 nm [24,25]. The Li et al. study demonstrated that silver nanoparticles typically exhibit an SPR band at around 370 nm, while silver nanoclusters show absorption at around 500 nm. As a well-defined absorption band around 500 nm can be observed in the UV-vis spectra (Figure 2B,C), this result strongly suggests the presence of silver nanoclusters. However, the absence of a distinct band at 370 nm indicates that the production of silver nanoparticles is negligible under our synthesis conditions [26].

The UV absorption at this wavelength clearly indicates that AgNCs have been successfully synthesized and incorporated into the polymer structure. Based on these results, it can be concluded that the synthesized AgNCs correspond to small clusters containing photo-reduced silver atoms, which are stabilized within the polymer [24,27].

**Figure 3:**
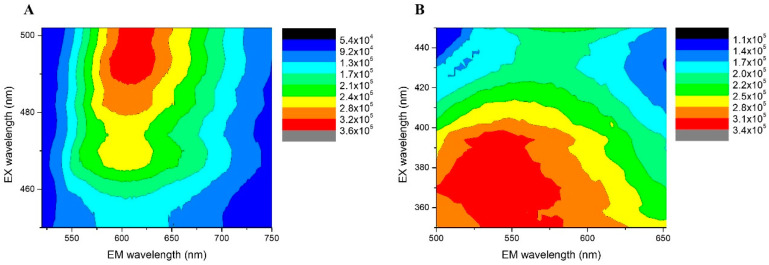
AgNCs emission–excitation matrices, recorded at different emission and excitation wavelengths. (**A**) Ag-PAA NCs; (**B**) Ag-PMAA NCs.

**Figure 4:**
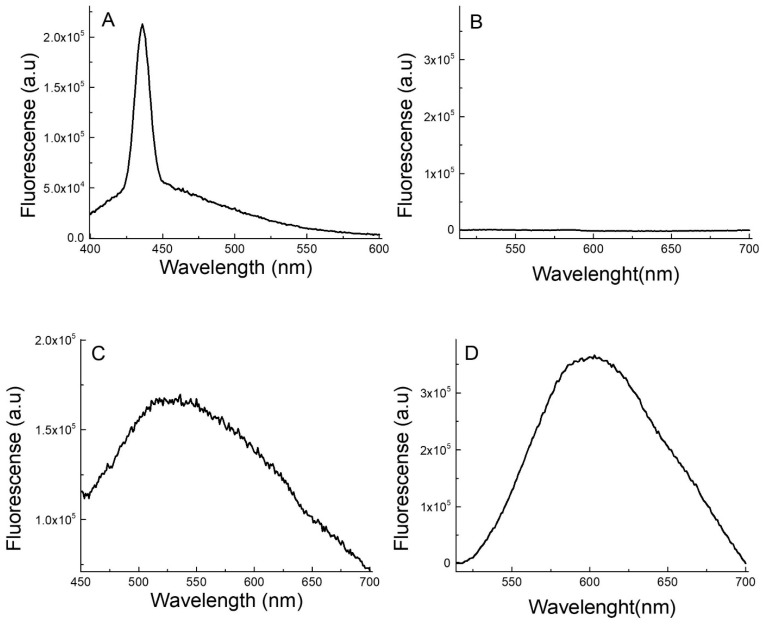
Fluorescence spectra of (**A**) PAA; (**B**) PMAA; (**C**) Ag-PAA NCs; and (**D**) Ag-PMAA NCs. Excitation wavelength (**A**,**C**): 380 nm. Ag-PAA NCs concentration: 100 µL of the AgNC stock solution in 2 mL of water. (**B**) Ag-PMAA NCs. Excitation wavelength (**B**,**D**): 490 nm. Ag-PMAA NC concentration: 100 µL of the AgNC stock solution in 2 mL of water.

AgNCs are known to be the only silver nanomaterial species that exhibit fluorescent transitions under UV radiation. To analyze and verify the presence of AgNCs, fluorescence spectroscopy experiments were carried out. The emission–excitation matrices (EEM) of the AgNCs provide fundamental insights into their photophysical behavior, as shown in Figure 3. Using EEM spectroscopy, the different species of AgNCs formed can be differentiated. The islands of the highest emission intensity (red areas) indicate the zones where the characteristic emission of each species is observed [28]. For Ag-PAA NCs, it can be noted that only the species of AgNCs predominated. The matrix reveals maximum emission in the range of 515 nm to 550 nm, when excited at wavelengths between 360 nm and 400 nm (Figure 3A). For Ag-PMAA NCs, two distinct emission areas with appreciable intensities were observed. This indicates the presence of two fluorescent species. Both species emitted light at a similar wavelength, probably due to their very close emission electronic states. Ag-PMAA NCs exhibited a maximum emission between 590 nm and 630 nm, with optimal excitation occurring between 480 nm and 500 nm (Figure 3B). The emission spectra of the polymers, Ag-PAA NCs and Ag-PMAA NCs, are shown in Figure 4. As can be seen, aqueous solutions of PAA (Figure 4A) and PMAA (Figure 4B) did not show any emissions in the range studied. In contrast, it can be observed that the Ag-PAA NCs (Figure 4C) showed strong emission with a maximum intensity at 525 nm, while the maximum band for the Ag-PMAA NCs (Figure 4D) was at 600 nm. Fluorescence was attributed to the size of AgNCs approaching the wavelength of Fermi electrons, their energy level structure becoming discrete, similar to that of individual molecules [29]. The main effect of quantum confinement in metal clusters is the appearance of a HOMO-LUMO energy-gap due to a discretization of the energy levels. Therefore, fluorescence confirmed the presence of AgNCs in the structure.

The position and shape of the emission band are influenced by several factors, including the type of metal group, size, the solvent used, and the stabilizing agent, among others [28,29,30]. Furthermore, the results indicated that the polymeric stabilization agents PAA and PMAA had significant effects on the optical properties of the AgNCs obtained, since the Ag-PAA NCs and the Ag-PMAA NCs exhibited fluorescence in different regions of the visible spectrum.

On the other hand, the formation and stability of the clusters can be easily monitored by observing the UV/vis or fluorescence spectra of the cluster solutions. Several authors have demonstrated that the optical properties of AgNCs are highly sensitive to pH, temperature, and solvents [30,31,32]. It is important to highlight that the type of stabilizer used plays a crucial role in determining the optical properties of AgNCs and can induce the formation of fluorescent clusters of different sizes. Yourston et al. demonstrated that using DNA molecules as stabilizers leads to the formation of two distinct AgNC species which produce green fluorescence and red fluorescence (<600 nm) [32]. Similarly, Nakal-Chidiac et al. showed that chitosan can be used to synthesize and stabilize AgNCs, emitting between 600 and 650 nm [33]. These findings demonstrate how different macromolecules can modulate the physicochemical properties of AgNCs while also acting as stabilizing agents, further influencing their optical behavior.

In this work, polymeric stabilizers such as PAA and PMAA played a fundamental role in modulating the optical properties of AgNCs and enhanced their stability. The interaction between carboxylate groups and silver atoms affects the electronic arrangement of the clusters, thereby determining their emission within the visible spectrum. The differences in fluorescence emission arise from variations in the rigidity of the polymeric matrix and the surrounding microenvironment, which modify the distribution of accessible electronic states and, consequently, the optical properties of the clusters. Furthermore, stabilizing polymers prevent aggregation, preserving the nanoscale size and ensuring long-term emission stability.

The AgNCs obtained using the proposed method maintained their fluorescent properties for at least 3 months when stored at 4 °C in the dark, indicating stability under these conditions. Additionally, no significant differences were observed in the temporal stability of AgNCs when comparing the effects of the stabilizing polymers, PAA, and PMAA. This underscores the potential and capability of both polymers as effective stabilizing agents for AgNCs. The temporal stability of AgNCs synthesized in this study was comparable to or better than that reported by other authors. For instance, Tegegne et al. [34] found that AgNCs stabilized on poly methyl hydro siloxane-modified filter paper (AgNC@SiO_2_/PMHS) remained stable for three months, while Pal et al. [35] reported that AgNCs stabilized with polyvinylpyrrolidone were stable for only two weeks. Similarly, Nagda et al. [36] synthesized DNA-encapsulated AgNCs that were stable for 24 days [37].

### 3.2. Antimicrobial Capacity of Ag-PAA NCs and Ag-PMAA NCs Against Staphylococcus aureus

**Figure 5:**
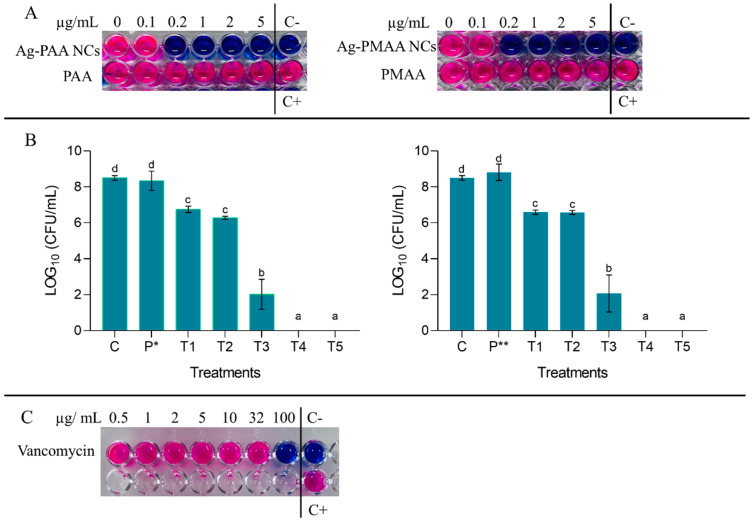
(**A**) Resazurin technique results and MIC and MBC determination after 3 h incubation. (**B**) Colony count of *S. aureus* after 3 h of incubation with different concentrations of AgNCs and the polymer. C (control: *S. aureus* + PBS); P* (pure PAA 5 μg/mL); P** (pure PMAA 5 μg/mL); T1 (AgNCs 0.1 μg/mL + *S. aureus*); T2 (AgNCs 0.2 μg/mL + *S. aureus*); T3 (AgNCs 1 μg/mL + *S. aureus*); T4 (AgNCs 2 μg/mL + *S. aureus*); and T5 (AgNCs 5 μg/mL + *S. aureus*). Different letters indicate significant differences between treatments based on ANOVA–Tukey Test (*p* < 0.0001). (**C**) Resazurin technique results with vancomycin and MIC determination after 3 h of incubation.

As a screening assay, a resazurin test was performed to calculate the MIC and MBC of Ag-PMAA NCs and Ag-PAA NCs. This rapid test indicated that the MIC for both AgNCs was 0.2 µg/mL, being the first AgNC dilution that remained blue after 3 h of incubation (Figure 5A). Although specific data on the MIC of AgNCs against *S. aureus* are limited, we found that other authors, after 24 h of incubation, obtained an MIC for silver NCs encapsulated in porous silica nanospheres significantly higher than 0.2 µg/mL (0.3 mg/mL) [38]. This suggests that the type of AgNC and its synthesis method could have a differential impact on the mechanism of action causing differences in MIC values. In addition, the MIC values found for Ag-PMAA NCs and Ag-PAA NCs were even lower than those obtained by other authors for silver nanoparticles against *S. aureus* (0.625 mg/mL in 24 h) [39], demonstrating a superior effect with respect to this type of nanomaterial. To compare the results obtained with a commonly used antibiotic for the treatment of *S. aureus*, we determined that the MIC for vancomycin was between 32 and 100 µg/mL (Figure 5C). Although vancomycin is a recommended antibiotic for treating severe infections caused by the MRSA strain, a dose of 20 µg/mL (maximal recommended dose) [7] was insufficient for the strain under study (*S. aureus*-ATCC 25923). According to the test performed, the MIC exceeded 32 µg/mL, which, based on the guidelines established by the CLSI and the SFM, indicated that the microorganism was resistant to this antibiotic.

Notably, the MIC achieved with the AgNCs synthesized in this work (0.2 µg/mL) was substantially lower than the MIC reported in the literature for vancomycin, highlighting their potential as an effective antimicrobial alternative.

In studies evaluating therapeutic failure, where increasing vancomycin MICs for *S. aureus* were analyzed, Rodríguez and Vesga [21] mentioned an MIC rated between 4 and 32 µg/mL, while Chander et al. [7] reported various MICs for strains of this microorganism ranging from 2 µg/mL to 20 µg/mL [7,21]. This finding not only suggests an alternative to using high concentrations of antibiotics but also opens the possibility of developing new treatment strategies that combine low doses of vancomycin with low doses of AgNCs.

For both AgNCs, the MBC was 2 µg/mL after 3 h of incubation, as it was the first AgNC dilution that showed zero colony count after the microdrop technique (Figure 5B). On the contrary, it can be observed that equal concentrations of PMAA and PAA did not display any effect on the microorganism since every well turned to pink due to an active bacterial metabolism capable of resazurin reduction (Figure 5A). Both AgNCs assayed had the same effect on the survival of *S. aureus*, displaying an effective antibacterial activity in a dose-dependent manner. In Figure 5B, the bars show that lower AgNC doses like 0.1 and 0.2 µg/mL (T1 and T2, respectively) inhibit pathogen development by generating a difference with the control by almost 2 LOG_10_ (CFU/mL), a result that was corroborated with the resazurin technique where an MIC was obtained at 0.2 µg/mL (Figure 5A). At 1 µg/mL, *S. aureus* survival was affected, notoriously presenting a difference of 6 LOG_10_ (CFU/mL) with the control; this effect was most remarkable at 2 and 5 µg/mL, where no colonies were detected (the viability of bacteria decreased to zero). It is important to note that the AgNCs developed in this work had a bactericidal effect at low doses if compared with AgNCs developed by Jin et al. [16], which did not show any effect on *S. aureus* using 50 µg/mL, and to eradicate *E. coli*, they needed 25 µg/mL. On the other hand, silver nanoparticles exhibited antibacterial activity against *E. coli* at a dose of 5 µg/mL [40], which was significantly higher than the 2 µg/mL required for our AgNCs to completely eliminate *S. aureus*. Additionally, it is important to note that Agnihotri et al. [40] conducted their assays with a bacterial concentration of 1 × 10^4^ CFU/mL, whereas our study used a concentration of *S. aureus* of 1 × 10^6^ CFU/mL. This suggests that AgNCs were not only effective at lower concentrations but also maintained their efficacy even when the pathogen load was high. Chen et al. [17] mentioned in their work that 6.4 µg/mL of AgNCs was needed to restrain *P. aeruginosa* viability at 90%. On the other hand, in the case of a conjugated tyrosine silver nanoparticle, the bibliography indicates that a concentration of 30 µg/mL was required to achieve the MBC for *S. aureus* [41].

It is notable that PAA or PMAA did not produce effects over microorganism survival. This could be given for its own properties (biocompatible-low cytotoxic polymers) [14] and confirmed the previous results with resazurin.

### 3.3. Effect of AgNCs on Bacterial Death Kinetics

**Figure 6:**
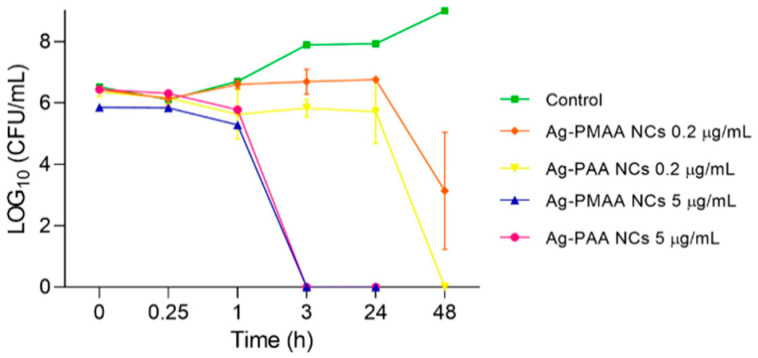
Effect of AgNCs on *S. aureus* death kinetics. *S. aureus* was incubated with 0.2 and 5 µL/mL of each nanocluster. Microdrop technique was performed to determinate the colony count at different times.

To find out in more detail how time influences the process of *S. aureus* growth inhibition and death by the AgNCs, a kinetic study was performed. The effect of each AgNC in two concentrations (0.2 and 5 µg/mL) was evaluated for 24 and 48 h (Figure 6). Figure 6, in line with the results above (Figure 5A,B), shows that the 0.2 µg/mL concentration of both AgNCs inhibited the microorganism development during 24 h; after this time, the amount of *S. aureus* started to decrease to a zero level for Ag-PAA NCs with a more moderated decrease for Ag-PMAA NCs at 48 h. The number of microorganisms tended to remain constant over time, reaching a maximum level at 24 h of 5.7 and 6.7 LOG_10_ (CFU/mL) for Ag-PMAA NCs and Ag-PAA NCs, respectively. Then, between 24 and 48 h, this concentration demonstrated a bactericidal effect by significantly reducing the bacterial population to 3.2 and 0 LOG_10_ (CFU/mL) for Ag-PMAA NCs and Ag-PAA NCs, respectively, in contrast with the control curve that increased to approximately 9 LOG_10_ (CFU/mL) at 48 h. Meanwhile, a 5 µg/mL concentration of both AgNCs triggered bacterial death at between 1 and 3 h of incubation, showing no viable cells at a further time. The results imply that 5 µg/mL of AgNCs is a bactericidal dose that achieves this result within a short period (3 h), and this could present a scenario where the bacteria, in the presence of this quantity of AgNCs, could not make changes fast enough to survive this situation. For example, Jin et al. [16] could not obtain MICs or MBCs with their AgNCs against *S. aureus* with their major concentration (50 µg/mL) after 24 h. In the case of conjugated tyrosine silver nanoparticles, to obtain an MBC of *S. aureus* was necessary at a concentration of 30 µg/mL for 18 h [41].

### 3.4. Hemocompatibility AgNCs “In Vitro”

**Figure 7:**
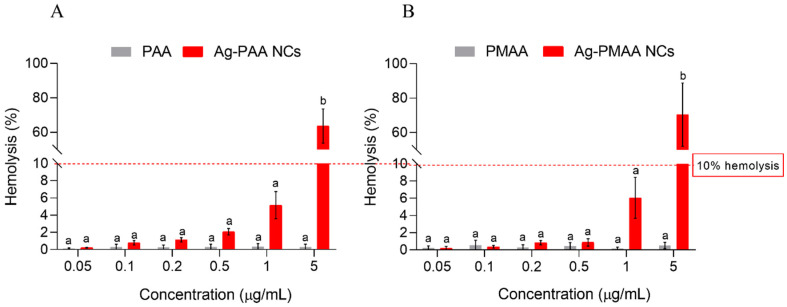
Biocompatibility evaluation of each nanocluster and their respective polymer. Hemolysis (%) quantification of Wistar rat red cells upon treating with different nanoclusters concentrations for 3 h. (**A**): Ag-PAA NCs contrasted with PAA. (**B**): Ag-PMAA NCs contrasted with PMAA. Different letters indicate significant differences between treatments based on ANOVA–Tukey Test (*p* < 0.0001).

Evaluating the hemolytic activity of compounds, including chemicals, drugs, or materials that may come into contact with blood, is a crucial step in the initial assessment of cytotoxicity. This method provides valuable insights into the potential mechanism of cell membrane disruption, which is a critical factor for applications in biological systems [42]. The hemolytic activity of each AgNC and their respective polymers was tested on red blood cells from female Wistar rats to evaluate cytotoxicity. None of the stabilizing polymers tested independently caused significant hemolysis when compared to the negative control (Figure 7, grey bars). Gautam et al. [43] reported an evaluation of PAA and PMAA toxicity in rats, where an oral administration of up to 2000 µg/mL of PAA or PMAA was conducted. Subsequent sampling of various organs revealed no significant alterations. PMAA, a polymer known for its high biocompatibility, has been utilized for decades in applications such as buccal cement and blood pumps, among others. Our hemolysis test results for the AgNCs indicated that at almost all tested concentrations, the hemolysis percentage remained below the acceptable hemocompatibility threshold of 10% [44]. However, hemolysis increased in a concentration-dependent manner, with a 5 µg/mL concentration showing the highest hemolytic activity (70% hemolysis), exceeding the hemocompatibility limit. Similar findings were reported by Darwish et al. [41] with silver nanoparticles (5 µg/mL) on human erythrocytes after 30 min of exposure. Salman et al. [45] demonstrated that free Ag⁺ ions, derived from AgNO_3_, displayed moderate activity against Staphylococcus aureus, with minimum inhibitory concentration and minimum bactericidal concentration values of 80 µg/mL. These findings align well with previously published data on S. aureus ATCC 12228, which reported MIC and MBC values of 31 µg/mL and 62 µg/mL, respectively [46]. It is important to note that the concentrations reported by Salman et al. and Oliveira Lima et al. were significantly higher than those associated with the clusters synthesized in our study using PAA and PMMA as stabilizers, which achieved an MIC of 0.2 µg/mL and MBC of 2 µg/mL. These results suggest that the synthesized clusters exhibited greater antimicrobial efficacy than silver ions. Additionally, previous research has indicated that AgNO₃ concentrations of 24 µg/mL can lead to a hemolytic rate exceeding 10% [47]. In contrast, the clusters synthesized in this investigation can be employed at concentrations of up to 1 µg/mL without causing significant hemolytic damage (<10%). Although concentrations lower than 1 µg/mL are recommended to prevent hemolytic damage, the silver nanoclusters demonstrated a remarkable improvement, exhibiting a more than 400-fold increase in the minimum inhibitory concentration compared to free silver ions. This significant enhancement highlights their potential for safer and more effective use in antimicrobial therapies.

These results are promising, as the hemolytic limit was not exceeded at the 1 µg/mL concentration, which, over 3 h, demonstrated a reduction of 6 LOG10 (CFU/mL) in viable cells compared to the control (Figure 5B). Concentrations over the 10% limit could have an effective application in inert surfaces as disinfectants or as coatings for medical devices to prevent *S. aureus* contamination [13].

## 4. Conclusions

In this work, AgNCs were successfully synthesized using an eco-friendly photoreduction method at pH 7, demonstrating reproducibility. Two different polymers, PAA and PMAA, were employed as stabilizers. Our findings revealed that the AgNCs optical properties are influenced by the stabilizer used, indicating significant stabilization and confinement effects provided by PAA and PMAA. The formation of AgNCs was confirmed through detailed physicochemical characterization. The use of PAA and PMAA as stabilizers not only facilitated the synthesis of AgNCs but also allowed for fine-tuning of their physicochemical properties. These findings provide valuable insights into the design of nanomaterials with controlled properties and sizes for potential biomedical applications.

The results obtained in this investigation showed that the AgNCs assayed presented antimicrobial effects in doses that were not cytotoxic to rat blood cells. There was no difference between both AgNCs in bactericidal efficacy (in time or in concentration).

It was possible to determine that 0.2 µg/mL of AgNCs did not eliminate the microorganism, but rather only prevented their multiplication in the early hours of treatment. However, after 48 h, this concentration showed a bactericidal tendency. Currently, the antibiotic in use, vancomycin, requires higher doses (15 to 20 mg/L) to be effective and, with the adverse effect of potential nephrotoxicity in higher doses to have a positive therapeutic effect. However, it was not effective against this strain of *S. aureus* ATCC 25923 due to the microorganism’s resistance. This opens the possibility of using AgNCs in two low applications or in a long-time treatment, like conventional antibiotics. Concentrations of up to 1 µg/mL led to microorganisms dying in 3 h and still maintained a biocompatibility with rat red cells.

While the stability of the AgNCs is sufficient for the intended application in this study, further improvements can be achieved by incorporating more robust ligands (thiols, phosphines), optimizing the polymer matrix with crosslinking agents, or fine-tuning synthesis parameters such as pH, ionic strength, and irradiation conditions. These strategies can enhance structural integrity and minimize degradation over time.

Future work should explore the behavior of AgNCs in complex biological environments and investigate their mechanism of action against *S. aureus*. Additionally, assessing their long-term biocompatibility and expanding their use in antimicrobial coatings for medical devices and wound dressings will be key to advancing their practical applications. Furthermore, future research should aim to broaden the understanding of AgNCs in biological settings, particularly by comparing their efficacy against Gram-negative bacteria such as *Pseudomonas aeruginosa*. Such studies would provide valuable insights into the broader antimicrobial potential of AgNCs and their mechanisms of action across different bacterial species. Based on the results of this study, since concentrations of AgNCs greater than 1 µg/mL show a hemolysis of 10%, we suggest that these AgNCs could be further investigated as topical agents for treating dermal infections caused *by S. aureus* or as disinfectants for inert surfaces and medical devices, such as catheters, to prevent biofilm formation.

## Data Availability

The dataset is available on request from the authors.
